# Association of Plasma Brain-Derived Tau With Functional Outcome After Ischemic Stroke

**DOI:** 10.1212/WNL.0000000000209129

**Published:** 2024-01-31

**Authors:** Tara M. Stanne, Fernando Gonzalez-Ortiz, Cecilia Brännmark, Katarina Jood, Thomas Karikari, Kaj Blennow, Christina Jern

**Affiliations:** From the Department of Laboratory Medicine (T.M.S., C.B., C.J.), Institute of Biomedicine, Sahlgrenska Academy, University of Gothenburg; Department of Clinical Genetics and Genomics (T.M.S., C.J.), Region Västra Götaland, Sahlgrenska University Hospital; Department of Psychiatry and Neurochemistry (F.G.-O., T.K., K.B.), Institute of Neuroscience and Physiology, Sahlgrenska Academy, University of Gothenburg; Clinical Neurochemistry Laboratory (F.G.-O., K.B.); Department of Research, Development, Education and Innovation (C.B.), Region Västra Götaland, Sahlgrenska University Hospital; Department of Clinical Neuroscience (K.J.), Institute of Neuroscience and Physiology, the Sahlgrenska Academy, University of Gothenburg; Department of Neurology (K.J.), Region Västra Götaland, Sahlgrenska University Hospital, Gothenburg, Sweden; and Department of Psychiatry (T.K.), University of Pittsburgh.

## Abstract

**Objectives:**

To investigate whether circulating acute-phase brain-derived tau (BD-tau) is associated with functional outcome after ischemic stroke.

**Methods:**

Plasma tau was measured by a novel assay that selectively quantifies BD-tau in the *Sahlgrenska Academy Study on Ischemic Stroke* (*SAHLSIS*), which includes adult cases with ischemic stroke and controls younger than 70 years, and in an independent cohort of adult cases of all ages (*SAHLSIS2*). Associations with unfavorable 3-month functional outcome (modified Rankin scale score >2) were analyzed by logistic regression. Various stratified and sensitivity analyses were performed, for example, by age, stroke severity, recanalization therapy, and etiologic subtype.

**Results:**

This study included 454 and 364 cases from the *SAHLSIS* and *SAHLSIS*2, with a median age of 58 and 68 years, respectively. Higher acute BD-tau concentrations were significantly associated with increased odds of unfavorable outcome after adjustment for age, sex, day of blood draw, and stroke severity (NIH stroke scale score) in both cohorts (OR per doubling of BD-tau: 2.9 [95% CI 2.2–3.7], *P* = 1 × 10^−15^ and 1.8 [1.5–2.2], *P* = 7 × 10^−9^, respectively). The association was consistent in the different stratified and sensitivity analyses.

**Discussion:**

BD-tau is a promising blood-based biomarker of ischemic stroke outcomes, and future studies in larger cohorts are warranted.

## Introduction

Tau is a microtubule-associated protein involved in mechanisms of plausible importance for ischemic brain injury including oxidative stress, excitotoxicity, apoptosis, and inflammation.^1^ CSF and blood-based total-tau (T-tau) are established biomarkers of neuronal and axonal damage in neurodegenerative diseases, and increased concentrations have also been reported in a few small studies on acute ischemic stroke.^1^ However, blood-based concentrations of T-tau do not correlate with T-tau concentrations in the CSF.^2^ We therefore recently developed an assay that selectively measures brain-derived tau (BD-tau) and not tau produced by peripheral tissues.^2^ We found that plasma/serum BD-tau outperforms T-tau as a biomarker for Alzheimer disease–type neurodegeneration^2^ and that increased BD-tau concentrations associated with unfavorable outcome after traumatic brain injury.^3^ Based on these findings, we hypothesize that circulating acute-phase BD-tau concentrations are associated with functional outcome after ischemic stroke.

## Methods

Anonymized data will be shared on reasonable request, provided data transfer agrees with EU legislation on the general data protection regulation and with decisions by the Ethical Review Board of Sweden and the University of Gothenburg, the latter which should be regulated in a data transfer agreement.

### Study Population

This study included cases and controls from the hospital-based observational longitudinal cohort study, the *Sahlgrenska Academy Study on Ischemic Stroke* (*SAHLSIS*), previously described and in the online supplement (eMethods, links.lww.com/WNL/D357).^4^ In brief, patients with first-ever or recurrent acute ischemic stroke aged 18–69 years were recruited between 1998 and 2003. For validation, a second observational longitudinal cohort study, the *SAHLSIS* phase *2* (*SAHLSIS2*),^5^ was used. This ongoing study includes first-ever or recurrent adult cases with acute stroke of all ages, and this study includes participants recruited during the period 2015–2020. For both cohorts*,* ischemic stroke was defined as an episode of focal brain dysfunction with acute onset, lasting >24 hours, and of presumed vascular cause with no signs of hemorrhage on neuroimaging. Participants were excluded if further evaluation showed another etiology than stroke. Etiologic stroke subtypes were classified according to the Trial of Org 10172 in Acute Stroke Treatment (TOAST) criteria^6^ with minor modifications as described.^7^

### Stroke Severity and Functional Outcome

In the *SAHLSIS*, the maximum stroke severity within the first 7 days of admission to the hospital was assessed by the Scandinavian Stroke Scale and converted to the NIH Stroke Scale (NIHSS) using an established algorithm.^8^ Of note, recruitment to the *SAHLSIS* took place before recanalization therapy was part of clinical routine treatment. In the *SAHLSIS2*, stroke severity was defined either as the NIHSS score at admission for patients who did not undergo recanalization therapy or 24 hours after recanalization therapy. Functional outcome was rated by the modified Rankin scale (mRS) at an in person 3-month follow-up visit in the *SAHLSIS*. In the *SAHLSIS2*, data on death and dependency 3 months after index stroke were retrieved from the Swedish national quality register Riks-Stroke and transformed into mRS scores as described.^9^ For both cohorts, the 3-month mRS scores were dichotomized into favorable (score 0–2) and unfavorable (score 3–6) outcomes.

### Blood Sampling and Protein Measurement

EDTA plasma was isolated after an overnight fast at inclusion (median 4 [IQR 3–6] and 2 [IQR 2–4] days after index stroke in the *SAHLSIS* and *SAHLSIS2*, respectively). BD-tau measurements were performed on the Simoa HDX platform (Quanterix, Lexington, MA) as described at the Clinical Neurochemistry Laboratory, Sahlgrenska University Hospital, Mölndal, Sweden.^2^ Acute serum levels of neurofilament light chain (NfL) were previously measured in the *SAHLSIS*.^10^ For details, see the online supplement (eMethods, links.lww.com/WNL/D357).

### Statistics

Binary logistic regressions were used to estimate associations with unfavorable outcome in univariable and multivariable analyses adjusted for age, sex, and day of blood draw (model 1) and stroke severity (model 2; model 1 + NIHSS score) in both cohorts separately. Data from both cohorts were merged for stratified analyses by etiologic stroke subtype, stroke severity (NIHSS score < vs ≥ 5), stroke location (right vs left hemisphere), and age (< vs ≥ median). For the *SAHLSIS2*, analyses were stratified based on intervention with recanalization therapy or not (i.e., intravenous thrombolysis and/or mechanical thrombectomy) and mechanical thrombectomy or not. Sensitivity analyses excluding patients with prestroke neurologic comorbidities, with prestroke functional disability, and younger than 70 years were also performed. Finally, the effect sizes of association with outcome for BD-tau and NfL (both Ln-transformed) were compared, and multiprotein regressions were performed to determine independent effects. Two-tailed *p* < 0.05 was considered significant.

### Standard Protocol Approvals

Written informed consent was obtained by all participants or next-of-kin. The *SAHLSIS* was approved by the Regional Ethics Review Board in Gothenburg, Sweden (469-99, T553-03, and 413-04, T665-07) and the *SAHLSIS2* by the Regional Ethics Review Board in Gothenburg (823-13, T1110-16) and the Swedish Ethics Review Authority (2022-00597-02).

## Results

**Figure 1:**
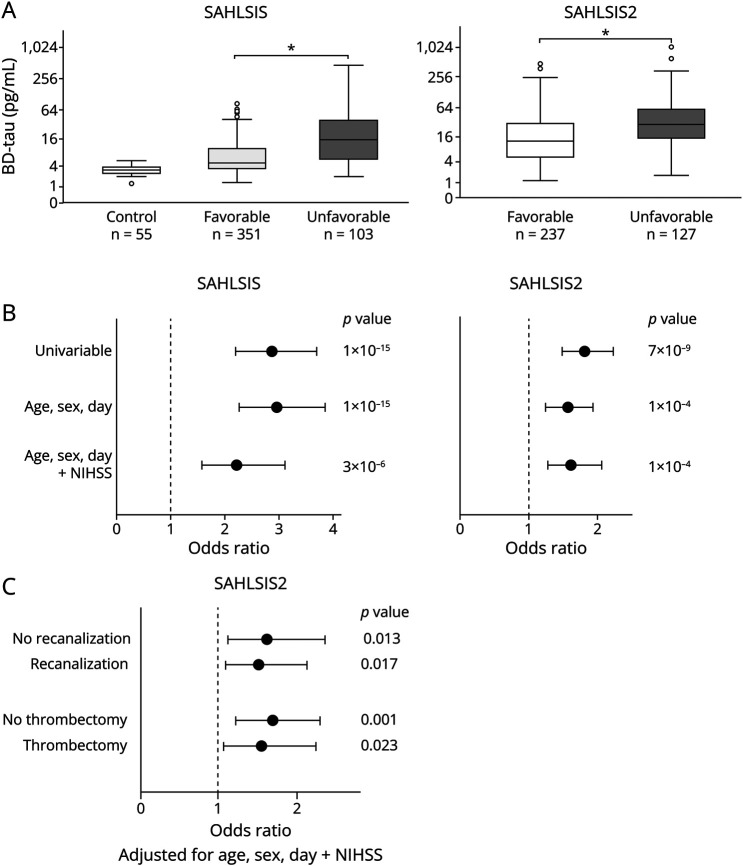
Plasma BD-Tau Concentrations Are Associated With 3-Month Functional Outcome in All Ischemic Stroke (A) Box plot of BD-tau in controls and in cases with ischemic stroke with favorable vs unfavorable outcome defined as modified Rankin scale (mRS) score 0–2 vs 3–6. *p < 0.001. (B–C) Forest plots of odds ratios for unfavorable outcome per doubling of BD-tau concentrations in (B) all ischemic stroke and C) stratified by intervention with or without recanalization therapy or thrombectomy.

In this study, 454 cases and 55 controls from the *SAHLSIS* and 364 cases from the *SAHLSIS*2 were included, and their baseline characteristics are summarized in Table 1. In the *SAHLSIS*, plasma BD-tau concentrations were higher in cases compared with those in controls (Table 1 and Figure 1A; *p*_*t*-test_ <0.001). In both cohorts, acute-phase BD-tau was higher in cases with unfavorable outcome compared with favorable outcome (Figure 1A; *p*_*t*-test_ <0.001), and this association was significant in multivariable regression analyses (Figure 1B; eTables 1–2, links.lww.com/WNL/D357). Median BD-tau concentrations were lower in the *SAHLSIS* compared with those in the *SAHLSIS2*, and this was mainly due to the lower age of *SAHLSIS* participants; for details, see the online supplement (eResults). In the *SAHLSIS2*, the association was significant when stratifying by recanalization therapy (Figure 1C).

**Table 1:** Baseline Characteristics for Controls and Cases With Ischemic Stroke in the *SAHLSIS* and *SAHLSIS2* and According to Functional Outcome Status as Determined by the Modified Rankin Scale (mRS) Score 0–2 vs 3–6 at 3-Month Follow-Up

	*SAHLSIS*				*SAHLSIS2*		
	Control	All ischemic stroke	3-mo outcome		All ischemic stroke	3-mo outcome	
			Favorable	Unfavorable		Favorable	Unfavorable
N	55	454	351	103	364	237	127
Age, median [IQR], y	58 [44–65]	58 [52–64]	58 [51–64]	60 [53–65]	68 [59–79]	65 [54–73]	78 [68–85]
Male sex, n (%)	37 (67)	303 (67)	228 (65)	75 (73)	231 (64)	166 (70)	65 (51)
Day of blood draw, median [IQR]	—	4 [3–6]	4 [3–6]	4 [2–6]	2 [2–4]	2 [1–3]	3 [2–6]
Hypertension, n (%)	16 (30)	270 (60)	207 (59)	64 (61)	171 (47)	90 (38)	81 (64)
Diabetes mellitus, n (%)	2 (4)	87 (19)	64 (18)	23 (22)	49 (13)	22 (9)	27 (21)
Smoker, n (%)	10 (18)	178 (39)	139 (40)	39 (38)	39 (11)	24 (10)	15 (12)
Stroke location, n (%)							
Right hemisphere	—	152 (34)	112 (32)	40 (39)	132 (46)	76 (42)	56 (54)
Left hemisphere		213 (48)	160 (47)	53 (52)	112 (39)	72 (40)	40 (38)
Brainstem or cerebellum		83 (18)	74 (21)	9 (9)	41 (14)	33 (18)	8 (8)
Intravenous thrombolysis, n (%)	—	3 (0.8)	1 (0.3)	2 (2)	112 (31)	77 (33)	35 (28)
Thrombectomy, n (%)	—	0	0	0	105 (29)	60 (25)	45 (35)
Stroke severity (NIHSS), median [IQR]^a^	—	2 [1–6]	2 [1–4]	12 [6–15]	5 [1–13]	3 [1–11]	9 [4–16]
24 h after recanalization therapy^b^, median [IQR]	—	—	—	—	3 [1–10]	1 [0–3]	9 [4–13]
Subtype: large artery atherosclerosis, n	—	54	38	16	44	21	23
Small artery occlusion, n	—	93	83	10	26	21	5
Cardioembolic stroke, n	—	64	42	22	102	61	41
Cryptogenic stroke, n^c^	—	131	107	24	56	42	14
Other determined cause, n^d^	—	37	21	16	25	18	7
Undetermined cause, n	—	75	60	15	58	38	20
Plasma BD-tau, median [IQR], pg/mL	3 [3–4]	5 [4–15]	5 [4–10]	16 [6–39]	16 [7–38]	12 [5–28]	28 [14–66]

a In the *SAHLSIS*, the maximum NIHSS score within the first 7 d of admission to the hospital was used; in the *SAHLSIS2*, the NIHSS was scored at admission (day 0) in all patients.

b In the *SAHLSIS2*, the subset of patients who underwent recanalization therapy (intravenous thrombolysis and/or mechanical thrombectomy) were also scored using the NIHSS 24 h after the procedure (day 1). For these patients, this was the NIHSS score used in regression models.

c No cause identified despite a complete workup.

d Incomplete evaluation or more than 1 identified cause.

**Figure 2:**
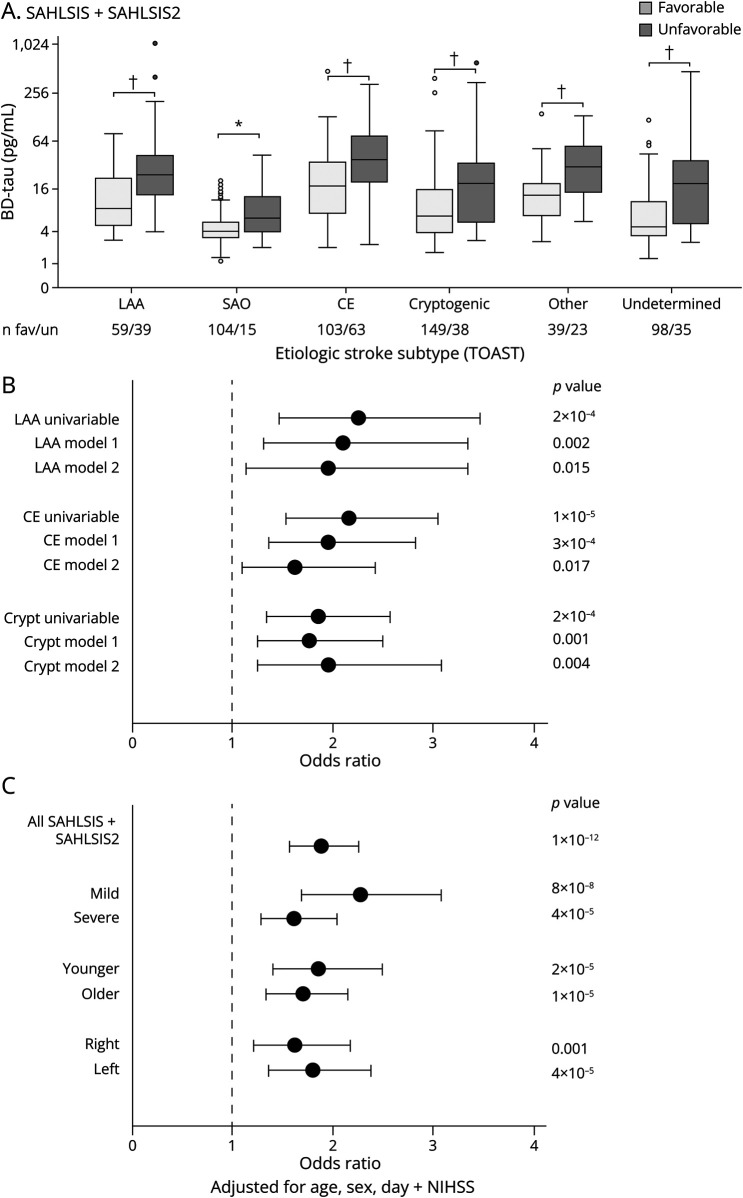
Plasma BD-Tau Concentrations Are Associated With 3-Month Functional Outcome Across Etiologic Stroke Subtypes and Other Strata in the Combined Cohort (A) Box plot of BD-tau in cases with ischemic stroke stratified by etiologic stroke subtype with favorable vs unfavorable outcome defined as modified Rankin scale (mRS) score 0–2 vs 3–6. *T* test *p < 0.05, †*p* < 0.001. (B–C) Forest plots of odds ratios for unfavorable outcome per doubling of BD-tau concentrations for (B) LAA, CE, and cryptogenic stroke. The SAO subtype had only 15 cases with unfavorable outcome and was not analyzed by multivariable analysis. (C) Stratified by stroke severity (mild vs more severe; NIHSS score < or ≥5); age (younger vs older; < or ≥ 62 years); stroke location (right hemisphere vs left hemisphere). CE = cardioembolic; Crypt = cryptogenic stroke; fav = favorable outcome; LAA = large artery atherosclerosis; Other = other determined causes; SAO = small artery occlusion; un = unfavorable outcome.

In the combined cohort, BD-tau concentrations were higher in patients with unfavorable outcomes across all stroke subtypes (Figure 2A), and for large artery atherosclerosis, cardioembolic, and cryptogenic stroke, the association was significant in multivariable analyses (Figure 2B; eTable 4, links.lww.com/WNL/D357). The association remained significant in a variety of sensitivity and stratified analyses (Figure 2C; eTables 1–3).

Finally, the effect size for BD-tau in the *SAHLSIS* was higher than for NfL, although the confidence intervals were overlapping; and when both proteins were included in multiprotein models, only BD-tau remained significant (eTable 4, links.lww.com/WNL/D357). BD-tau was also more weakly correlated to day of blood draw compared with NfL (*r* 0.11 vs 0.30; *p* 0.02 and <0.001, respectively).

## Discussion

We found an association between elevated acute-phase plasma concentrations of the novel blood-based biomarker BD-tau and unfavorable functional outcome after ischemic stroke that was independent of both age and stroke severity (a proxy for infarct size), the 2 strongest known predictors of poststroke outcome,^11^ in 2 independent cohorts. Consistent results were observed in stratified analyses according to etiologic stroke subtype, stroke severity, stroke location, age, and recanalization therapy groups, indicating that BD-tau may serve as a biomarker of outcome in most ischemic stroke subgroups. Compared with the neuroaxonal damage marker NfL, previously shown by us and others to associate with poststroke functional outcome,^10,12,13^ acute-phase BD-tau was more weakly correlated to day of blood draw and showed stronger association with outcome.

The principal strength of this study is the inclusion of consecutive hospital-based stroke cases in 2 independent clinical cohorts with different case mixes. Limitations to consider include that both cohorts were recruited from the same area of Sweden and might not be generalizable to populations of other races or ethnicities, the proportion of mild strokes was relatively high, we cannot rule out confounding due to early recurrences or new but clinically silent cerebral ischemia, and the day of blood sampling was not standardized.

To conclude, the current results suggest that plasma BD-tau has potential as an accessible blood-based biomarker of ischemic stroke outcome. Future studies in larger stroke cohorts are warranted to validate the present findings as are studies with repeated blood draws to examine the optimal day of sampling for outcome prediction.

## Appendix Authors

**Table:**

Name	Location	Contribution
Tara M. Stanne, PhD	Department of Laboratory Medicine, Institute of Biomedicine, Sahlgrenska Academy, University of Gothenburg; Department of Clinical Genetics and Genomics, Region Västra Götaland, Sahlgrenska University Hospital, Gothenburg, Sweden	Drafting/revision of the article for content, including medical writing for content; study concept or design; and analysis or interpretation of data
Fernando Gonzalez-Ortiz, MD, MSc	Department of Psychiatry and Neurochemistry, Institute of Neuroscience and Physiology, Sahlgrenska Academy, University of Gothenburg; Clinical Neurochemistry Laboratory, Region Västra Götaland, Sahlgrenska University Hospital, Gothenburg, Sweden	Drafting/revision of the article for content, including medical writing for content; major role in the acquisition of data; and analysis or interpretation of data
Cecilia Brännmark, MD, PhD	Department of Laboratory Medicine, Institute of Biomedicine, Sahlgrenska Academy, University of Gothenburg; Department of Research, Development, Education and Innovation, Region Västra Götaland, Sahlgrenska University Hospital, Gothenburg, Sweden	Drafting/revision of the article for content, including medical writing for content
Katarina Jood, MD, PhD	Department of Clinical Neuroscience, Institute of Neuroscience and Physiology, the Sahlgrenska Academy, University of Gothenburg; Department of Neurology, Region Västra Götaland, Sahlgrenska University Hospital, Gothenburg, Sweden	Drafting/revision of the article for content, including medical writing for content; study concept or design
Thomas Karikari, PhD	Department of Psychiatry and Neurochemistry, Institute of Neuroscience and Physiology, Sahlgrenska Academy, University of Gothenburg, Sweden; Department of Psychiatry, University of Pittsburgh	Drafting/revision of the article for content, including medical writing for content
Kaj Blennow, MD, PhD	Department of Psychiatry and Neurochemistry, Institute of Neuroscience and Physiology, Sahlgrenska Academy, University of Gothenburg; Clinical Neurochemistry Laboratory, Region Västra Götaland, Sahlgrenska University Hospital, Gothenburg, Sweden	Drafting/revision of the article for content, including medical writing for content; study concept or design; and analysis or interpretation of data
Christina Jern, MD, PhD	Department of Laboratory Medicine, Institute of Biomedicine, Sahlgrenska Academy, University of Gothenburg; Department of Clinical Genetics and Genomics, Region Västra Götaland, Sahlgrenska University Hospital, Gothenburg, Sweden	Drafting/revision of the article for content, including medical writing for content; major role in the acquisition of data; and analysis or interpretation of data

## References

[1] Chen X, Jiang H. Tau as a potential therapeutic target for ischemic stroke. Aging. 2019;11(24):12827-12843. doi:10.18632/aging.102547 31841442 PMC6949092

[2] Gonzalez-Ortiz F, Turton M, Kac PR, et al. Brain-derived tau: a novel blood-based biomarker for Alzheimer's disease-type neurodegeneration. Brain. 2023;146(3):1152-1165. doi:10.1093/brain/awac407 36572122 PMC9976981

[3] Gonzalez-Ortiz F, Dulewicz M, Ashton NJ, et al. Association of serum brain-derived tau with clinical outcome and longitudinal change in patients with severe traumatic brain injury. JAMA Netw Open. 2023;6(7):e2321554. doi:10.1093/brain/awac407 37399012 PMC10318474

[4] Jood K, Ladenvall C, Rosengren A, Blomstrand C, Jern C. Family history in ischemic stroke before 70 years of age: the Sahlgrenska Academy Study on Ischemic Stroke. Stroke. 2005;36(7):1383-1387. doi:10.1161/01.STR.0000169944.46025.09 15933254

[5] Dorvall M, Pedersen A, Dumanski JP, et al. Mosaic loss of chromosome Y is associated with functional outcome after ischemic stroke. Stroke. 2023;54(9):2434-2437. doi:10.1161/STROKEAHA.123.043551 37465995 PMC10453343

[6] Adams HP Jr, Bendixen BH, Kappelle LJ, et al. Classification of subtype of acute ischemic stroke. Definitions for use in a multicenter clinical trial. TOAST. Trial of Org 10172 in Acute Stroke Treatment. Stroke. 1993;24(1):35-41. doi:10.1161/01.str.24.1.35 7678184

[7] Olsson S, Holmegaard L, Jood K, et al. Genetic variation within the interleukin-1 gene cluster and ischemic stroke. Stroke. 2012;43(9):2278-2282. doi:10.1161/STROKEAHA.111.647446 22744645

[8] Ali K, Cheek E, Sills S, Crome P, Roffe C. Development of a conversion factor to facilitate comparison of National Institute of Health Stroke Scale Scores with Scandinavian Stroke Scale Scores. Cerebrovasc Dis. 2007;24(6):509-515. doi:10.1159/000110420 17971629

[9] Eriksson M, Appelros P, Norrving B, Terent A, Stegmayr B. Assessment of functional outcome in a national quality register for acute stroke: can simple self-reported items be transformed into the modified Rankin Scale? Stroke. 2007;38(4):1384-1386. doi:10.1161/01.STR.0000260102.97954.9c 17322093

[10] Pedersen A, Stanne TM, Nilsson S, et al. Circulating neurofilament light in ischemic stroke: temporal profile and outcome prediction. J Neurol. 2019;266(11):2796-2806. doi:10.1007/s00415-019-09477-9 31375988 PMC6803587

[11] Rost NS, Bottle A, Lee JM, et al Global Comparators Stroke GOAL collaborators. Stroke severity is a crucial predictor of outcome: an international prospective validation study. J Am Heart Assoc. 2016;5(1):e002433. doi:10.1161/JAHA.115.002433 26796252 PMC4859362

[12] Tiedt S, Duering M, Barro C, et al. Serum neurofilament light: a biomarker of neuroaxonal injury after ischemic stroke. Neurology. 2018;91(14):e1338-e1347. doi:10.1212/WNL.0000000000006282 30217937

[13] Uphaus T, Bittner S, Gröschel S, et al. NfL (Neurofilament light chain) levels as a predictive marker for long-term outcome after ischemic stroke. Stroke. 2019;50(11):3077-3084. doi:10.1161/STROKEAHA.119.026410 31537188

